# Utilization of serology for the diagnosis of suspected Lyme borreliosis in Denmark: Survey of patients seen in general practice

**DOI:** 10.1186/1471-2334-10-317

**Published:** 2010-11-01

**Authors:** Ram B Dessau, Jette M Bangsborg, Tove Ejlertsen, Sigurdur Skarphedinsson, Henrik C Schønheyder

**Affiliations:** 1 Department of Clinical Microbiology, Næstved Hospital, Region Zealand, Næstved, Denmark; 2 Department of Clinical Microbiology, Herlev Hospital, Copenhagen University Hospital, Herlev, Denmark; 3 Department of Clinical Microbiology, Aalborg Hospital, Aarhus University Hospital, Aalborg, Denmark; 4 Department of Clinical Microbiology, Odense University Hospital, Odense, Denmark

## Abstract

**Background:**

Serological testing for Lyme borreliosis (LB) is frequently requested by general practitioners for patients with a wide variety of symptoms.

**Methods:**

A survey was performed in order to characterize test utilization and clinical features of patients investigated for serum antibodies to *Borrelia burgdorferi *sensu lato. During one calendar year a questionnaire was sent to the general practitioners who had ordered LB serology from patients in three Danish counties (population 1.5 million inhabitants). Testing was done with a commercial ELISA assay with purified flagella antigen from a Danish strain of *B. afzelii*.

**Results:**

A total of 4,664 patients were tested. The IgM and IgG seropositivity rates were 9.2% and 3.3%, respectively. Questionnaires from 2,643 (57%) patients were available for analysis. Erythema migrans (EM) was suspected in 38% of patients, Lyme arthritis/disseminated disease in 23% and early neuroborreliosis in 13%. Age 0-15 years and suspected EM were significant predictors of IgM seropositivity, whereas suspected acrodermatitis was a predictor of IgG seropositivity. LB was suspected in 646 patients with arthritis, but only 2.3% were IgG seropositive. This is comparable to the level of seropositivity in the background population indicating that Lyme arthritis is a rare entity in Denmark, and the low pretest probability should alert general practitioners to the possibility of false positive LB serology. Significant predictors for treating the patient were a reported tick bite and suspected EM.

**Conclusions:**

A detailed description of the utilization of serology for Lyme borreliosis with rates of seropositivity according to clinical symptoms is presented. Low rates of seropositivity in certain patient groups indicate a low pretest probability and there is a notable risk of false positive results. 38% of all patients tested were suspected of EM, although this is not a recommended indication due to a low sensitivity of serological testing.

## Background

Lyme borreliosis (LB) is a diagnosis frequently considered in general practice and a large number of samples are submitted to the diagnostic laboratory for serological testing. The use of antibody testing in populations with low pretest probability is cause for concern[1,2]. The aim of this study was to describe the utilization of serological testing for *Borrelia burgdorferi *sensu lato including IgG and IgM serum antibodies in general practice in Denmark. Information on the clinical spectrum of patients and the rates of positive and negative serological results reported in this study may assist the general practitioners in assessing the laboratory reports. To our knowledge, data about the utilization of LB serology in general practice and the clinical characteristics of both seropositive and seronegative patients has not been published previously.

## Methods

A survey was performed in three Danish counties (Copenhagen, Funen and North Jutland, total population 1.47 million) of patients with LB serology being requested by a general practitioner or a practicing specialist. In each county LB serology was performed in only one diagnostic laboratory located at the main hospital. Thus, the study was based on the entire population in the three counties. During one full year from March 2001 to February 2002 a questionnaire was included with the LB serology report sent to general practitioners and practising specialists. 694 patients were excluded because they were subsequently referred to a hospital department and tested for intrathecal antibody production. The doctors were asked to classify the clinical manifestation of each patient according to the seven EUCALB case-definitions, which were printed on the backside of the questionnaire[3]. Indication of "various other symptoms" and "no current symptoms" was also an option.

### Laboratory methods

All three laboratories used the same commercial assay for measurement of IgG and IgM serum antibodies (IDEIA™, Oxoid, Cambridge, UK) which is based on purified flagella antigen from a Danish strain of *B. afzelii*. The assay was performed according the protocol from the manufacturer. Grey zone results were considered negative. The cut-off values of the test were from the outset adjusted by the manufacturer to be 98% specific in a healthy Danish blood-donor population for both IgM and IgG antibodies[4]. The specificity did not change over time as confirmed by recent testing of healthy Danish blood donors living in an endemic area in Zealand[5]. According to the literature, the diagnostic sensitivities for IgG/IgM antibodies are 36%/48% in erythema migrans (EM), 77%/57% in early neuroborreliosis (NB) and 100%/12% in acrodermatitis chronica atrophicans[4,6-8]. The results of all serological samples were retrieved from the departments' laboratory information systems. Patients were identified by the unique Danish civil registration number and each sample was coded with a unique number and an identifier for the general practitioner.

### Statistical methods

From patients with repeat samples only the first sample was included in the analysis. The rate of seropositivity was chosen as dependent variable in statistical analyses of the diagnostic yield in different groups of patients according to clinical symptoms, season, reported tick bite, county, sex and age groups. The chi-square test was used for 2 × 2 tables (p values < 0.05 were considered significant). 95% confidence intervals (CI) on the binomial probability were calculated by the Wilson method. A generalized additive model (i.e. a non-linear model) was used for logistic regression due to the nonlinear characteristics of the seasonal variation[9]. All analyses were performed with "R" statistical software[10]. See appendix for further details.

### Ethics

The Danish Registry Board (record 2001-41-0617) approved the study according to guidelines for registry-based research.

## Results

A total of 4,664 patients seen in general practice had LB serology performed.

Thus, the incidence of testing for LB was 267 per 100,000 per year. 427 (9.2%, CI 8.4-10.0%, Wilson) patients were IgM antibody positive and 155 (3.3%, CI 2.8-3.9%, Wilson) were IgG antibody positive.

### Response rate

2,643 (57%) questionnaires were returned by 627 general practitioners. A possible selection bias was evaluated by logistic regression with data from the laboratory information systems. Compared to the other age groups the response rate was lower for the age group 16 to 50 years old (54%, OR 0.83, CI 0.73-0.94). The response rate was related neither to sex nor season. The IgG seropositivity rate was 3.4% for the patients included versus 3.2% for those not included (chi-square p = 0.66). However, the IgM seropositivity rate was 10.4% for the patients included and 7.5% for those not included (chi-square p = 0.0013).

### Clinical manifestations in patients tested for LB

A rash (suspected to be EM) was seen in 1,011 (38%) of the patients (Table 1). Lyme arthritis was suspected in 603 (23%) patients and neurological symptoms were recorded in 340 (13%) patients.

**Table 1:** Main results and rates of seropositivity.

Variable	Category	N	IgG positive		IgM positive		Treated	
			
			%	OR(95%CI)	%	OR(95%CI)	%	OR(95%CI)
	Total	2643	3.4		10.4		21.1	^1^N = 2569

Age group	0-15 years	195	3.1	0.60(0.25-1.44)	**21.0**	**2.29(1.52-3.47)**	24.3	0.84(0.55-1.3)
	16-50 years	1162	**1.8**	**0.39(0.23-0.65)**	9.0	1.01(0.76-1.34)	18.3	0.96(0.76-1.2)
	>50 years	1286	4.8	reference	10.0	reference	23.2	reference

Sex	Female	1509	3.1	reference	10.1	reference	21.5	reference
	Male	1134	3.7	1.23(0.79-1.91)	10.7	1.00(0.77-1.30)	20.6	0.90(0.72-1.1)

Reported tick bite	No bite	1468	3.3	reference	9.2	reference	12.0	reference
	^2^"Insect bite"	346	**2.0**	**0.42(0.18-0.97)**	**7.2**	**0.50(0.32-0.79)**	26.8	1.36(0.98-1.9)
	Tick bite	829	4.0	0.88(0.54-1.44)	13.6	1.05(0.79-1.47)	**34.9**	**2.9(2.2-3.7)**

^3^Exposure	Low	167	1.2	0.39(0.09-1.70)	6.0	0.81(0.40-1.63)	21.1	0.54(0.27-1.1)
	Normal	2153	3.2	reference	10.7	reference	22.1	reference
	High	323	5.6	1.59(0.89-2.83)	10.2	0.95(0.63-1.43)	7.9	0.90(0.64-1.3)

**Suspected Lyme disease manifestation**^**4**^								

Rash		1011	4.7	1.82(0.95-3.47)	**17.2**	**3.30(2.22-4.89)**	**44.9**	**7.2(5.0-10)**
Early neuroborreliosis		340	2.4	0.88(0.36-2.15)	6.2	0.95(0.55-1.63)	7.7	0.72(0.44-1.2)
Lymphocytoma		28	0		7.1	0.65(0.13-3.13)	17.9	1.80(0.62-5.3)
Carditis		14	0		21.4	3.66(0.93-14.35)	23.1	1.90(0.41-8.7)
Arthritis		603	2.3	0.79(0.37-1.66)	6.6	0.97(0.63-1.52)	**8.4**	**0.63(0.42-0.94)**
Acrodermatitis		67	**10.4**	**3.20(1.24-8.25)**	10.4	1.42(0.61-3.28)	13.6	0.93(0.42-2.1)
Chronic Neuroborreliosis		130	1.5	0.54(0.25-1.18)	5.4	0.87(0.45-1.69)	5.5	0.57(0.24-1.35)
Various		330	1.5	0.52(0.19-1.44)	6.7	0.91(0.53-1.56)	7.8	0.63(0.38-1.1)
No current clinical symptoms		367	3.8	1.22(0.61-2.46)	9.5	1.21(0.78-1.87)	**8.3**	**0.38(0.24-0.60)**

Characteristics of 2,643 patients tested for Borrelia antibodies. The table includes the rates of seropositivity for IgG and IgM, the percent of patients treated with antibiotics, and the corresponding odds ratios for the three multivariate logistic regression models.

Statistically significant odds ratios (OR) with 95% Confidence Intervals (CI) not including "one" are highlighted with bold

^1^Missing information about treatment from 74 patients.

^2^This option was called "other insect bite" on the questionnaire.

^3^Exposure was proposed as High if the patient was a farmer, forest worker and hunter or other patients with similar outdoor activities. Low exposure was proposed as a "person in rare contact with nature". All others were considered "normal".

^4^More than one symptom was registered for some patients. Categories for each symptom are "present" with "absent" as reference. The table has been shortened by omitting the% IgG positive,% IgM positive and% Treated for the patients when the respective symptom(s) are absent. These percentages were similar to the total average% positive/treated at the top of the table.

### Rates of seropositivity and treatment

**Figure 1:**
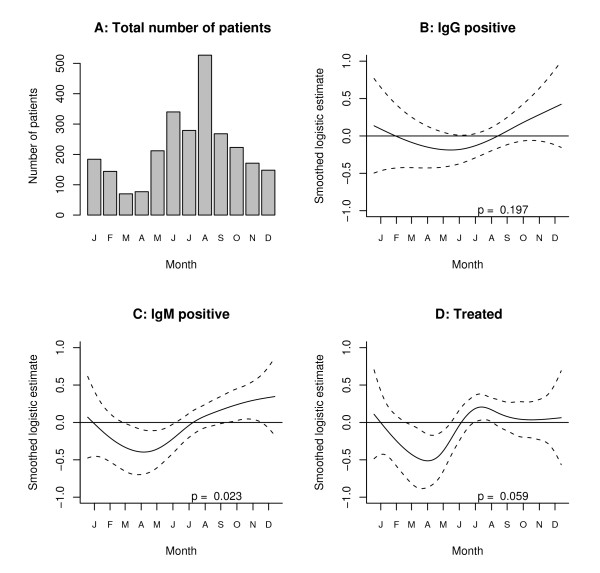
**Seasonal characteristics of test utilization and treatment for Lyme borreliosis**. Four graphs A, B, C, D in one figure. The smoothed logistic regression curve with 2 standard errors above and below the estimate shows the seasonal variation in the proportion of patients who are IgG antibody positive, IgM antibody positive or treated for Lyme disease. The horizontal line at zero indicates the mean number. Months of the year are abbreviated by the first letter.

IgM and IgG seropositivity rates and rates of treatment for LB are shown in Table 1 and Figure 1 for the 2,643 patients with clinical data. IgM seropositivity rates were found to be elevated in children (21%, OR 2.3) and patients with a rash (17.2%, OR 3.3). The IgG seropositivity rate was high in the 67 patients suspected of acrodermatitis (10.4%, OR 3.6). A low rate of IgG antibody (2%, OR 0.4) and IgM antibody (7.2%, OR 0.5) was found in patients reporting an "insect bite" instead of a tick bite. The lowest IgG seropositivity rate (1.8%, OR 0.4) was found in patients aged 16-50 years. In patients with a rash 19.4% was IgG or IgM antibody positive.

There was a distinct seasonal variation in testing activity (Figure 1A) with a trough of 70 patients in March and a peak of 527 patients in August. However, the non-linear analysis showed fairly constant rates of seropositivity throughout the year with a tendency towards higher rates in the autumn and a trough in April; this variation was statistically significant only for IgM antibodies (Figure 1C).

Antibiotic treatment for LB was prescribed to 21.1% of all the patients. Higher treatment rates were found in patients with tick bites (34.9%, OR 2.9) and patients with a rash (44.9%, OR 7.2).

### Tick bite and rash

In patients with a rash the rate of IgM seropositivity was the same (19%) whether they reported a tick bite or not (Table 2). However, in patients with a rash and a tick bite 56% were treated compared to 36% of the group with no bite reported. In patients where "other insect bite" was indicated only 10% were IgM positive.

**Table 2:** Details of seropositivity in patients with a rash and a tick bite.

	Bite lesion	Number	%IgM positive	%treated
No rash	No bite	1079	6	3
	Other insect bite	137	4	7
	Tick bite	416	8	14

Rash present	No bite	389	19	36
	Other insect bite	209	10	40
	Tick bite	413	19	56

Three way table of the number of patients and% IgM seropositive according to the combination of a reported rash and bite lesion.

## Discussion

This study provides information on the clinical manifestations of patients with LB serology testing in general practice in Denmark. The rates of seropositivity presented thus represent a mixture of patients with active Borrelia infection, immunological memory or non-specific cross-reactivity. In more than one third of the patients EM was suspected and these patients received antibiotic therapy also when antibody findings were negative. This is in agreement with the perception that EM with a characteristic presentation or history of an expanding rash is a distinct clinical entity and that laboratory testing is unnecessary and of limited utility[1,11].

The average rate of IgG antibodies was low (3.4%) with a tendency to be even lower in patients suspected of Lyme arthritis (2.3%) and chronic NB (1.5%) which comes very close to the expected background seropositivity of 2%. This indicates a large proportion of false positive results and a pretest probability lower than recommended[12].

High IgM seropositivity rates were found in patients with suspected EM and in children (17.2 and 21.0%, respectively). A high IgG seropositivity rate (10.4%) was found in patients with suspected acrodermatitis.

### Validity of the survey

The response rate for the clinical survey was 57% and there is a possibility of selection bias. However, we had access to descriptive data on all patients through the laboratory information systems of the participating departments. It was noted that compared to patients not included there were fewer patients aged 16-49 years in the study group and the rate of IgM seropositivity was slightly higher. Whether this reflected a selection bias for certain clinical manifestations is not known and overestimation of the seropositivity rate in some of the clinical subgroups is possible. From a methodological point of view, it would be desirable if the questionnaire had been completed during the first consultation with the patient, this being so before the serological results became available. Still, we believe that classification of patients was not materially influenced by the laboratory report. Altogether, there seems to have been a preference for inclusion of IgM positive patients and the seropositivity rates and OR for IgM antibody results shown in Table 1 are likely to be marginally too high.

### Seropositivity rates and the clinical variables

The overall rate of seropositivity in our study compares well with another Danish study of 897 consecutive samples obtained during the winter months, December to April, where 9.9% were IgM and 2.0% were IgG positive using the same flagella based assay[13].

In the present study 829 (31%) of the tested patients reported a tick-bite. This is a much lower rate than seen in a Swedish survey of patients with reported LB. In this study 79% of the patients were aware of a tick bite preceding the onset of symptoms[14]. This disparity may be explained by the difference in populations studied, as only patients with a reported diagnosis of LB were included in the Swedish study. Our study confirms that a history of a tick bite is not only a frequent cause for serological testing, but also an important motivation for the prescription of antibiotic therapy. Still, it has been shown that only about 0.5% of tick bites lead to clinical LB[15].

Tick bites were a fairly common complaint in our study (13.6% of patients). This was also shown in a Dutch study that reported an annual number of 4.6 patients with tick bites compared to only 0.9 patient with an EM diagnosis per doctor (average practice population 2430 people)[16]. Furthermore, reports from the USA indicate that a tick bite may lead to costly and unnecessary serologic testing or prophylactic antibiotic therapy[17,18].

When our patients reported a tick bite it was most likely due to Ixodes ricinus and not a non specified "insect-bite" as other arthropods in Denmark do not remain attached to the skin for a longer period and has to be removed. This could explain why patients with "other insect bites" were less frequently found to be IgM and IgG antibody positive (Tables 1 and 2). More than one third (38%) of the tests were requested from patients with a rash, although this is not generally recommended due to the relatively low sensitivity of antibody testing in this group[3,7,19] and the clinical diagnosis is considered to be reliable [20,21]. Treatment was given to 44.9% of patients with a rash. The rate of seropositivity for either IgG or IgM antibodies was 19.4%, indicating that the decision to treat was based on a sensible clinical evaluation.

It is recommended to perform a lumbar puncture when NB is suspected[3,19,22]. In our study 340 patients with suspected early NB and 130 with suspected chronic NB were not further investigated with a lumbar puncture and testing for intrathecal antibody production. As about 20% of patients with early NB may be seronegative[23], some cases might have been overlooked. However, we cannot preclude that some patients may have been referred to neurological evaluation, but a specialist did not deem testing for intrathecal antibody production relevant after evaluation.

Concerning possible stage 3 LB a high sensitivity of IgG antibodies is expected. Judged on the rate of IgG seropositivity in the group of patients with suspected acrodermatitis the doctors were conscious of this entity. But when using serology to support the diagnosis of Lyme arthritis the main problem was the low prevalence of this disease in the tested population. Only 2.3% of patients suspected of arthritis were found to be IgG positive in the present study (Table 1). This rate is close to the level of reactivity in the healthy adult population. Thus, in some subgroups there are few true cases of LB and the rate of seropositivity is close to the healthy background population. It must also to be expected that some patients with vague clinical symptoms are tested to rule out the diagnosis of LB rather than to confirm it. In addition, the slight or non significant variation of the monthly rates of seropositivity for both IgM and IgG support the notion, that many patients had non-typical clinical manifestations. The seasonal variation of seropositivity would have been more pronounced if more straightforward cases of EM had been included.

It is possible that patients with clinical disease other than LB may develop cross-reacting antibodies more frequently than the healthy donor population, which was used to adjust the specificity of the IgM antibody ELISA. For all subgroups of patients in the present study the rate of IgM seropositivity was at least 5.4%. This may indicate that the finding of a solitary IgM positive test in patients with non-distinct symptoms should be interpreted with caution.

The selection of patients is assumed to be the important factor for the rates of seropositivity and the results obtained in this Danish study may not be directly generalized to other countries, where the epidemiology of LB, clinical practice, diagnostic methods, and recommendations may differ. Nevertheless, low rates of seropositivity, and thus low pretest probability, in certain patient groups may be a significant problem also in other countries. It has been recommended that the pretest probability of LB should be no less than 0.20[1,12]. We are not aware of studies assessing whether this recommendation is fulfilled in clinical practice. As shown in this study, such a high rate of pretest probability is probably not realistic when diagnosing e.g. Lyme arthritis. Also contributing to the low rate of IgG seropositivity could be that the test has a lower sensitivity in a consecutive stream of routine patients compared to studies on selected well defined cases, as these studies may have inherent problems with representativeness[5].

It is generally recommended to use a "confirmatory" assay such as a Western blot (WB) in a two-tier sequence in order to improve specificity[1,22]. However, WB is difficult to standardize[24,25]and it has never been evaluated on consecutive clinical samples, whether this approach indeed improves the classification of patients with and without active LB. The alternative strategy is instead to use an assay with a strict cut-off allowing only 2% false positives as assessed in the local blood donor population for IgM and IgG antibody, respectively. Healthy blood donors are assumed to represent the lowest possible antibody reactivity in the population. The flagella assay is designed in accordance with this strategy and maintains an acceptable sensitivity. If using WB together with the flagella assay the cut-off would need adjustment, otherwise the sensitivity would become unacceptably low. No diagnostic laboratory in Denmark uses WB as a second line assay to modify the specificity[26].

To our knowledge, this study is the first allowing a crude assessment of the rate of false positive serological tests in patients who are tested but do not have active LB. We define an IgM or IgG antibody test to be falsely positive if it occurs in a patient who does not have any other indication of active LB. The maximum number of false positives in excess of the healthy donor population may be crudely estimated by subtracting 2% from rates of seropositivity in Table 1. For example in the group of patients with arthritis 3.4% are IgM positive in excess of the healthy blood donors. The low rate of IgG seropositivity shows that Lyme arthritis is indeed rare. This implies that the lowest total estimate of the specificity for the IgM antibody test could be 94.6 percent instead of the expected 98%.

False positive IgM results are documented to be common in patients with Epstein-Barr virus infection (54%) and less so in patients with cytomegalovirus infection (8%) [27]. These rates do, however, not pertain to patients tested by general practitioners in the routine clinical setting, and it would be misleading to include them in a calculation of specificity. Moreover, these viral infections do not follow the same seasonal trend as LB where the large bulk of tests are carried out between May and October (Figure 1A). By extensive analysis of the current data we found no subgroup to fall below 5-6% in IgM seropositivity during winter months (data not shown) so the false rate could be estimated up to a maximum of 5-6%. Concerning IgG antibodies there is no indication that the specificity of IgG differs from about 98%.

An interesting finding in this study is a nearly threefold difference in the rate of IgM (10.4%) seropositivity compared to IgG (3.4%). This difference has not been found in neither blood donors or from patients with Lyme borreliosis [4,6-8]. One hypothesis for the high frequency of IgM responses could be that many patients with short duration of clinical Lyme borreliosis are tested. Another hypothesis could be non-specific reactions in patients with other diseases. The answer is most likely a combination of both. The seasonal distribution of the frequencies of samples and IgM responses support the hypothesis that true early Borrelia infections contributes, at least partially, to the high IgM response.

The above interpretation of the rates of seropositivity above rests on the important assumptions that low rates of seropositivity indicate fewer and high rates indicate more patients with active infection due to Borrelia burgdorferi even when some of the tested patients may have non-specific antibody responses.

Still, the results obtained in this questionnaire-based study may have important implications for the guidelines provided to general practitioners for interpretation of serological tests for LB.

## Conclusions

Low rates of seropositivity in certain patient groups indicate a low pretest probability and accordingly there is a notable risk of false positive results. 38% of all patients were tested for suspected EM, although this is not recommended due to the low sensitivity of the serological assay.

## Abbreviations

CI: confidence interval; ELISA: Enzyme linked immunosorbent assay; EM: Erythema migrans; LB: Lyme borreliosis; NB: Neuroborreliosis; OR: odds ratio.

## Competing interests

The authors declare that they have no competing interests.

## Authors' contributions

RBD conceived the study, performed data management, the statistical analyses and drafted the manuscript. JMB, TE, SS and HCS have been extensively involved in the design of the project, data collection, data analysis and preparation of several versions of the manuscript. All authors read and approved the final manuscript.

## Appendix

### Statistical issues

#### Choice of model

The regression models were used as a data mining/discovery tool to search for groups of patients with higher rates of seropositivity.

#### Model descriptions

Four multivariate models were built for the study:

Analysis of the complete dataset with response to survey as dependent variable was modelled using a generalized logistic regression model where Pi is the probability of being included in the clinical survey:

logit(Pi)=0.299+0.152*Age(0,15)−0.168*Age(15,50)+SEX(male)*0.032

The standard error is 0.12 for the children and 0.06 for the other estimates. The seasonal component was chosen as the first half of the year (January to June) compared with the second half (July to December).

Analysis of the seropositivity for IgG, IgM (= Positive) and Treatment (= Yes) as dependent variables was modelled using a logistic regression models with a smooth function for the seasonal variation. The Generalized additive model (GAM) developed by Simon Wood and implemented in R [8] was chosen, as it is possible combine both categorical and continuous variables into one multivariate model. GAM is useful as non-parametric smoothers may be fitted to the data without requiring specification of a particular mathematical model to describe the non-linearity. This type of model is an extension of the generalized linear model and gives an objective (non user chosen) description of the non-linear data. This eliminates the need to choose stratification into i.e. administrative units like calendar months which are more indirectly related to the seasonal behaviour of ticks or people. The syntax for the multivariate GAM-model (IgM as an example) was:

$$\begin{array}{l} \text{Model1IgM}<-\text{gam}(\text{IgMpos}\sim \text{AGE}+\text{SEX}+\text{TICK2}+\text{EXPOSURE2}\\ \;+\text{RASH}+\text{EARLYNB}+\text{LYMFOCYTOM}+\text{CARDIT}\\ \;+\text{ARTHRITIS}+\text{ACA}+\text{KNB}+\text{VARIOUS}+\text{NOCLINIC}+\text{s}\left(\text{PDATO4},\text{bs}="\text{cr}"\right),\\ \;\text{data}=\text{klin2},\text{family}=\text{binomial}) \end{array}$$

The variables (named as in the database) are in the order corresponding to Table 1.

The syntax for the smooth term for the seasonal variable was s(PDATO4,bs = "cr")[8]. The results of the model are stored in an R-object named "Model1IgM". The p-value for the smooth term (Figure 1. B, C and 1D) indicate whether the curved regression line departs significantly from zero. The seasonal variable were entered into the model as "day of the year" numbered from 1 to 365 starting with January 1st according to the date of the specimen, thus using the smallest available unit for analysis. This does not correspond literally to the data collection, which started from February to April 2001 and ended in February to March 2002 with some differences between the three laboratories. This means that the data from February to April are the combination of data from the same months in 2001 and 2002. In our experience the low total number of samples received during winter and the rate of seropositivity is similar from year to year (data not shown).

The GAM-models for IgG and treatment as dependent variables were identical, except that there was no positive IgG result among the few patients suspected of carditis or lymphocytoma.

The age distribution of the rates of seropositivity was initially examined by a non-linear GAM model prior to choosing the more simple age stratification in three groups. The age distribution of Lyme disease/seropositivity with a higher frequency in children and older adults has also been described previously [13].

127 patients had more than one symptom recorded (e.g. both a rash and various symptoms). This issue has not been considered separately. However, interaction terms (* instead of +) between different variables were tested in the model resulting in nonsignificant regression coefficients with large standard errors.

## Pre-publication history

The pre-publication history for this paper can be accessed here:

http://www.biomedcentral.com/1471-2334/10/317/prepub
